# Use of susceptibility-weighted imaging in assessing ischemic penumbra: A case report

**DOI:** 10.1097/MD.0000000000006091

**Published:** 2017-02-10

**Authors:** Xiujuan Wu, Song Luo, Ying Wang, Yang Chen, Jun Liu, Jing Bai, Jiachun Feng, Hongliang Zhang

**Affiliations:** a Neuroscience Center, Department of Neurology, The First Hospital of Jilin University; b Department of Neurosurgery, the Second Hospital of Jilin University, Jilin University, Changchun, China.

**Keywords:** diffusion-weighted imaging, ischemia penumbra, perfusion-weighted imaging, susceptibility-weighted imaging

## Abstract

**Rationale:**

The ischemic penumbra assessment is essential for the subsequent therapy and prediction of evolution in patients with acute ischemic infraction. Although controversial as a perfect equivalence to penumbra, perfusion-weighted imaging (PWI)-diffusion-weighted imaging (DWI) mismatch may predict the response to thrombolysis. Due to the reliance of PWI on contrast agents, noninvasive alternatives remain an unmet need.

**Patient concerns:**

We reported a 65-year-old man complained of paroxysmal hemiplegia of his right limbs and anepia for 2 days, whereas the symptoms lasted for about 12 hours when he admitted to the hospital.

**Diagnosis:**

We diagnosed it as acute ischemic stroke caused by the left middle cerebral artery stenosis.

**Interventions:**

Susceptibility-weighted imaging (SWI), multimodal magnetic resonance imaging (MRI) work-up which includes conventional MRI sequences (T1WI, T2WI, and FLAIR), DWI, PWI.

**Outcomes:**

His DWI-SWI mismatch was comparable to that of DWI-PWI at admission, suggesting that DWI-SWI could predict ischemic penumbra in patient with acute infarction. He refused the digital subtraction angiography examination or stenting, and he was treated with aspirin, atorvastain, and supportive treatment. The patient received a reexamination of the conventional MRI and SWI 11 days later. Expansion of the infarction in the affected MCA territory resulted from the penumbra indicated by the mismatch between DWI-SWI.

**Lessons:**

SWI can be used as a noninvasive alternative to evaluate the ischemic penumbra. Besides, SWI can provide perfusion information comparable to PWI and SWI is sufficient to identify occlusive arteries.

## 1 Introduction

In the hypoperfused brain tissue, for example, the increased oxygen extraction fraction via the metabolic compensation mechanism results in the elevation of the ratio of deoxyhemoglobin to oxyhemoglobin of the venous system of the brain, which contributes to the magnetic susceptibility difference between veins and the surrounding ischemic brain tissue.^[1,2]^ Deoxyhemoglobin which has a magnetic susceptibility effect usually generates hypointensity on susceptibility-weighted imaging (SWI) due to the presence of unpaired electrons resulting in rapid dephasing of proton spins.^[1]^ Assessment of ischemic penumbra is essential for initiating thrombolytic therapy and for predicting evolution or deterioration in patients with acute ischemic infraction. Although controversial as a perfect equivalence to penumbra, perfusion-weighted imaging (PWI)-diffusion-weighted imaging (DWI) mismatch is considered to predict the response to thrombolysis. Due to the reliance on contrast agents in PWI, noninvasive alternatives remain an unmet need. SWI is capable of providing additional clinical information complementary to conventional magnetic resonance imaging (MRI) sequences. SWI has been extensively used in the evaluation of various neurological disorders, including traumatic brain injury, neoplasms, intracranial calcification, vascular malformations cerebral venous thrombosis, spontaneous hemorrhagic transformation or postthrombolytic hemorrhagic transformation, intraarterial clot, microbleeds or iron deposition associated with neurodegenerative disorders, etc.^[1,2]^ In addition, due to its ability to mirror the pathophysiology of the acute ischemic stroke, SWI has recently attracted attention in the identification of penumbra, as well as in the prediction of the evolution or the deterioration of acute ischemic infarction.^[2–4]^

Kesavadas et al^[5]^ have found that the increased susceptibility due to the elevated deoxyhemoglobin-to-oxyhemoglobin ratio led to the visualization of prominent vein over the affected cerebral hemisphere on SWI. Interestingly, the SWI-DWI and the mean transit time (MTT)-DWI mismatches were found to be associated with deterioration of acute ischemic stroke.^[6]^ In this regard, SWI might illustrate misery perfusion with a compensatory increase of the oxygen extraction, and SWI is potentially a noninvasive alternative to PWI in predicting penumbra.^[6]^ We herein report a case in which SWI was used to evaluate penumbra.

## 2 Consent

Written informed consent was gained from the patient for the publication of the case report and accompanying images. The ethical approval of this study was waived by the ethics committee of The First Hospital of Jilin University because it was a case report.

## 3 Case report

**Figure 1:**
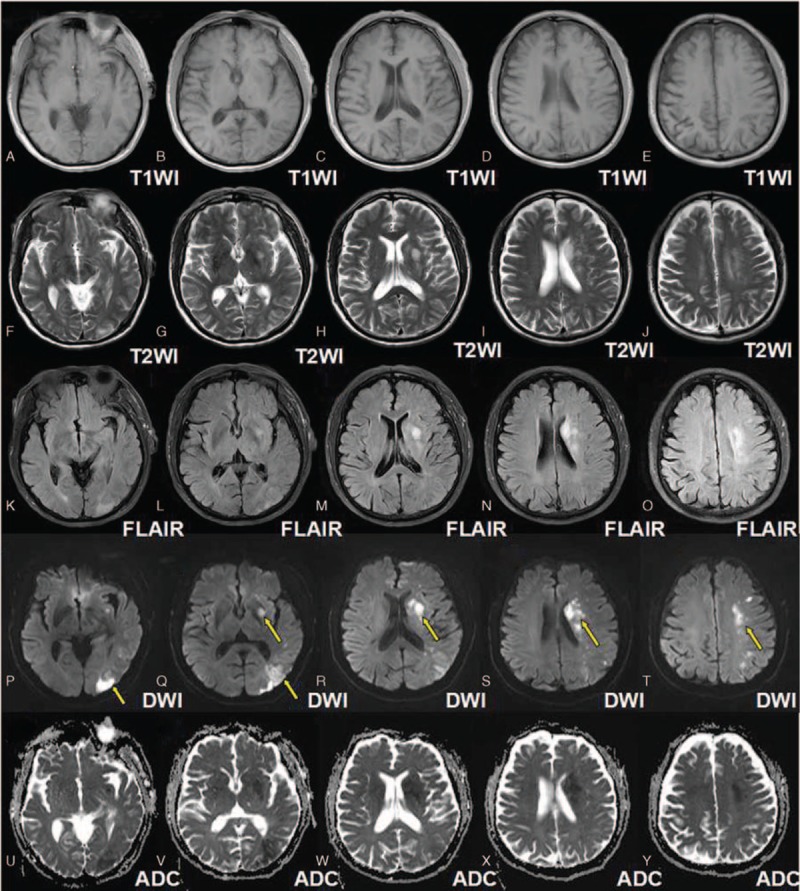
Magnetic resonance imaging panels of the representative case. A–E were T1WI which showed no obvious abnormalities, while E–J and K–O were respectively the T2WI and the FLAIR image which revealed the hyper intense signals predominately involved in the regions of internal capsule, corona radiata, and centrum semiovale. P–T showed more extensive involvements (arrows) on DWI while U–Y the ADC maps consistent with DWI finding. ADC = apparent diffusion coefficient, DWI = diffusion-weighted imaging, FLAIR = fluid attenuated inversion recovery, T1WI = T1-weighted image.

**Figure 2:**
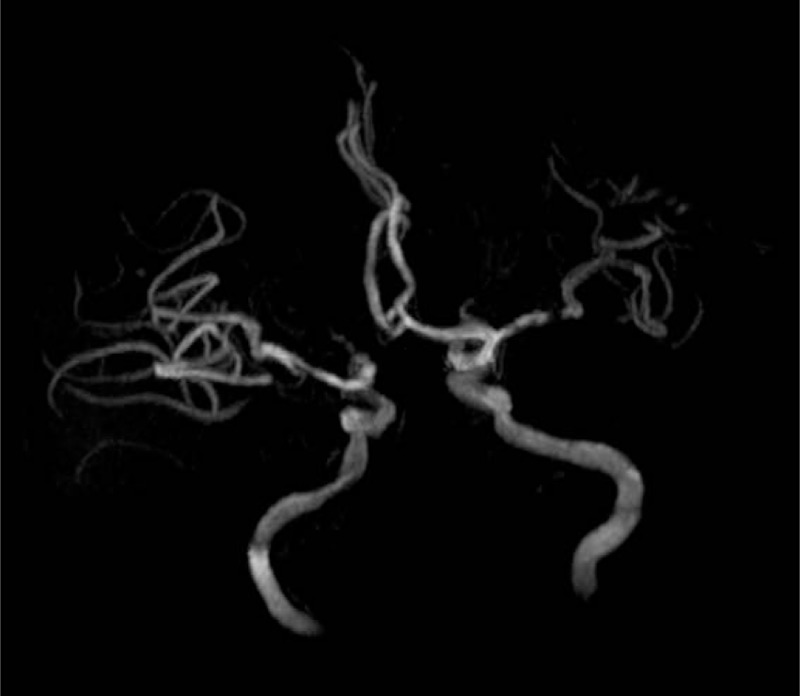
Stenosed middle cerebral artery. MRA revealed a stenosis in the left MCA. MRA = magnetic resonance angiography.

**Figure 3:**
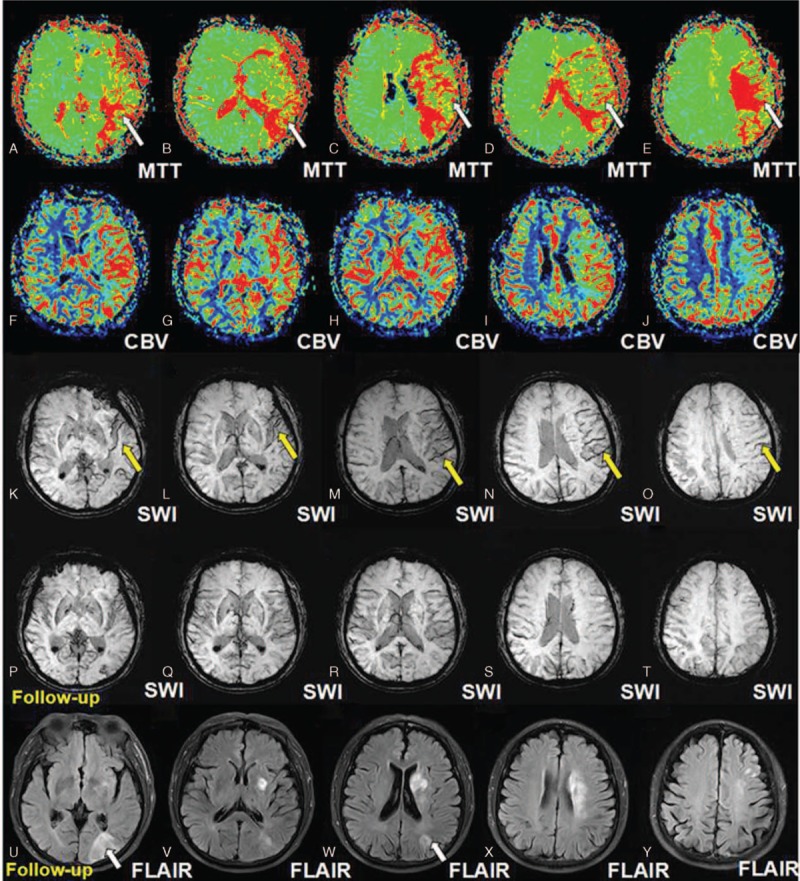
Perfusion weighted imaging and susceptibility weighted imaging and follow-up imaging. A–E (arrows) indicated the more prolonged MTT on PWI of the affected MCA and F–J showed elevated CBV of the affected MCA. In addition, the K–O (arrows) revealed ACVS or PV on SWI in the affected MCA territory, which disappeared in the follow-up SWI, as revealed by P–T. The mismatch between DWI-PWI and DWI-SWI, indicating the ischemic penumbra, was comparable. The expansion of the infarction resulting from the penumbra in the affected MCA territory, as shown on FLAIR image during follow-up indicated by the mismatch between DWI-SWI which was comparable with that of DWI-PWI. ACVS = asymmetrical cortical vessel sign, CBV = cerebral blood volume, DWI = diffusion weighted imaging, FLAIR = fluid attenuated inversion recovery, MTT = mean transit time, PV = prominent vein, PWI = perfusion weighted imaging, SWI = susceptibility-weighted imaging.

A 65-year-old man complained of paroxysmal hemiplegia of his right limbs and anepia for 2 days, whereas the symptoms lasted for about 12 hours when he admitted to the Department of Neurology in the First Hospital of Jilin University. MRI was performed with a Siemens 3T Trio Tim scanner (Siemens Medical, Erlangen, Germany) by using a standard head coil. The following MRI sequences were obtained in the axial plane with the following parameters: spin echo (SE) T1WI (time to repetition [TR]: 440 ms, time to echo [TE]: 2.5 ms); fast spin echo (FSE) T2WI (TR: 50,000 ms, TE 93 ms); FSE inversion recovery (IR) FLAIR: (TR: 8000 ms, TE: 93 ms, TI: 2371.5 ms); DWI (TR: 93 ms, TE: 3800 ms); MRA (TR: 25 ms, TE: 4.6 ms, slice thickness: 0.9 mm); PWI (TR: 2220 ms, TE 45 ms, slice thickness: 5 mm); and SWI (TR: 30 ms, TE 20 ms, slice thickness: 2 mm, flip angle 15°, iPAT factor of 2 and a matrix of 177 × 256 pixels). All these sequences were done with a slice thickness of 6 mm, unless otherwise specified, a gap of 1.2 mm, field of view (FOV) of 256 mm × 256 mm, incentive number of 1. No obvious abnormalities were revealed on T1WI (Fig. 1A–E) while hyperintense signals were found on T2WI (Fig. 1F–J) and FLAIR (Fig. 1K–O) which predominately involved the regions of internal capsule, corona radiata, and centrum semiovale. Extensive involvements were found on DWI (Fig. 1P–T) and ADC (Fig. 1U–Y) accompanied with severe stenosis of the left middle cerebral artery (MCA) as revealed by the MRA (Fig. 2). Prolonged MTT (Fig. 3A–E) and elevated CBV (Fig. 3F–J) on PWI, asymmetrical cortical vessel sign and prominent vein (Fig. 3K-O) on SWI was found in the left MCA territory. The DWI-SWI mismatch was comparable to that of DWI-PWI, suggesting that DWI-SWI could predict ischemic penumbra in patient with acute infarction. He refused the digital subtraction angiography examination or stenting, and he was treated with aspirin, atorvastain, and supportive treatment. The patient received a reexamination of the conventional MRI and SWI 11 days later. Expansion of the infarction in the affected MCA territory resulted from the penumbra indicated by the mismatch between DWI-SWI. Thus, the DWI-SWI mismatch is comparable with that of DWI-MTT in predicting ischemic penumbra.

## 4 Discussion

SWI has attracted increasing attention in identifying the ischemic penumbra and in predicting the prognosis of patients with acute ischemic infarction. From the study, we found that SWI was potential to evaluate the ischemic penumbra and could provide perfusion information comparable with PWI.

Penumbra represents conditionally reversible ischemic tissue which relies on the early and adequate reperfusion in patients with ischemic stroke. Thus far, DWI-PWI mismatch remains a widely accepted approach to represent penumbra which may benefit from thrombolytic treatment in patients with acute ischemic stroke.^[7]^ SWI, as a high-spatial-resolution 3-dimensional gradient-echo MR techniques, is highly susceptible to paramagnetic substances and has demonstrated advantages over conventional gradient-echo T2∗-weighted imaging.^[8]^ This patient did not receive thrombolytic therapy or stenting for the stenosed MCA, and expansion of the infarction in the affected MCA territory resulting from the penumbra was found during hospitalization. However, our study disclosed that the DWI-SWI mismatch in predicting penumbra was comparable with that of DWI-PWI. The finding was consistent with Kao et al study,^[6]^ which reported that the ASPECTS values were correlated with those of MTT. SWI is a noninvasive, contrast-independent, and less time-consuming alternative to PWI, and SWI-DWI mismatch may help identify penumbra and to select patients indicated for thrombolytic therapy.

However, Verma et al^[9]^ found that although SWI is helpful in detecting tissue at risk whereas cannot replace PWI. The reason might be that MTT detects more ill-perfused areas than SWI, especially in good collateralized subjects.^[10]^ The incidence of the hypointense vessels on SWI in patients with acute ischemic stroke varied among studies, and they questioned SWI as an alternative.^[9–11]^

In summary, SWI can be used as a noninvasive alternative to evaluate the ischemic penumbra. Besides, SWI can provide perfusion information comparable to PWI and SWI is sufficient to identify occlusive arteries.

## Acknowledgments

The authors thank National Natural Science Foundation of China (No. 81271293, 81271294, and 81241147) for the support.
